# Preparation of an MMT-Modified Hyperbranched Adsorbent and Its Application in the Selective Adsorption of Pb(II)

**DOI:** 10.3390/polym18121535

**Published:** 2026-06-20

**Authors:** Wei Gong, Shitong Xie, Meilan Li, Qiang Xie, Yinyin Zhou, Yutong Sun, Guochun Zhang

**Affiliations:** 1 Shaanxi Key Laboratory of Comprehensive Utilization of Tailings Resources, Shangluo University, Shangluo 726000, Chinaxsthh01@163.com (S.X.);; 2 Shaanxi Engineering Research Center for Mineral Resources Clean & Efficient Conversion and New Materials, Shangluo University, Shangluo 726000, China; 3 Chengdu Institute of Organic Chemistry, Chinese Academy of Sciences, Chengdu 610041, China

**Keywords:** hyperbranched, adsorbent, selective adsorption, heavy metal ions, Pb(II)

## Abstract

The P(IA-HBP-AA-AM)/MMT composite was successfully synthesized via in situ polymerization and characterized using FTIR, XRD, TGA, and other techniques. The material was then applied as an adsorbent for the removal of heavy metals from simulated mining-contaminated water (prepared based on the typical ionic composition of real mining wastewater). Static adsorption experiments revealed that P(IA-HBP-AA-AM)/MMT composite could efficiently remove Pb(II) from contaminated water, and the adsorption behavior was well described by the pseudo-second-order kinetic model and the Langmuir isotherm model. Thermodynamic analysis indicated that the adsorption of Pb(II) onto the P(IA-HBP-AA-AM)/MMT composite was an endothermic and spontaneous process. At pH = 4.5 and T = 45 °C, the maximum adsorption capacity obtained from model fitting was 249.38 mg/g. The material exhibited strong selectivity for Pb(II), even in the presence of competing metal ions such as Cd(II), Zn(II), Al(III), Fe(III), K(I), and Na(I). Moreover, after five adsorption–desorption cycles, it still retained approximately 90% of its Pb(II) removal efficiency. Furthermore, dynamic adsorption experiments showed that the saturation adsorption capacity of Pb(II) reached 178.7 mg/g, with a column utilization efficiency of approximately 41%. These findings demonstrate the promising potential of P(IA-HBP-AA-AM)/MMT composite for the removal of Pb(II) from mining-contaminated water.

## 1. Introduction

The exploitation and utilization of mineral resources have contributed to socioeconomic growth, facilitated infrastructure development in mining areas, and improved the living standards of local residents [1]. Nevertheless, the long-term adoption of irrational and unscientific extensive development practices has led to a range of ecological and environmental problems, such as soil erosion, surface subsidence, vegetation destruction, and heavy metal contamination of water and soil [2,3,4]. According to statistical data, the total discharge of heavy metals into wastewater in China in 2022 was approximately 120.7 tons, with heavy-metal-contaminated land accounting for about 70% of the total polluted land [5]. Lead (Pb) is a typical heavy metal pollutant that can accumulate in organisms and enter the human body through drinking water, crop irrigation, and food consumption. At concentrations exceeding 100 μg/L in wastewater, it poses significant risks to human health, fishery production, and agricultural irrigation. Furthermore, even exposure to low concentrations of lead is toxic and can lead to liver failure, brain damage, anemia, and neurological dysfunction [6,7]. According to World Health Organization (WHO) standards, the permissible concentration limit for Pb(II) in wastewater is generally 0.1–1 mg/L. In this context, the development of economic and efficient methods for treating Pb(II) in contaminated water around mining areas has become a research hotspot in environmental science.

In recent years, the main techniques for treating heavy metal pollution in contaminated water have included chemical precipitation, membrane separation, ion exchange, and adsorption [8,9,10,11,12,13,14,15]. Chemical precipitation involves adding precipitants to convert heavy metal ions into insoluble compounds. While this method is simple to operate and amenable to scale-up, it often introduces secondary pollution and increases the complexity of subsequent treatment [16]. Membrane separation, by contrast, relies on the selective rejection of semipermeable membranes to remove heavy metals, offering both low secondary pollution and high removal efficiency. Nevertheless, the large-scale application of membrane separation is constrained by the high cost of membrane materials, limited permeation flux, and short service life of membrane modules [17]. Ion exchange resins operate via reversible exchange reactions between their functional groups and heavy metal ions [18]. Although these resins can be regenerated and reused, the regeneration process often demands expensive chemical reagents and considerable energy input [19]. In contrast, adsorption has become a widely adopted technique for the removal of environmental pollutants, owing to its strong selective adsorption capacity, simple operation, low material cost, broad applicability, and excellent reusability [20,21,22,23].

Currently, polymer adsorbents have garnered growing research interest owing to their high structural designability and favorable functional tunability [24,25,26,27]. These adsorbents typically contain active functional groups, such as -NH_2_, -SH, and -COOH, in their molecular structures. Through mechanisms including electrostatic adsorption, coordination complexation, and ion exchange, these groups can specifically interact with heavy metal ions in water, enabling efficient removal [28]. Nevertheless, most existing polymer adsorbents feature linear molecular structures. In linear polymers, molecular chains are prone to entanglement via van der Waals forces or hydrogen bonding, which buries or shields numerous internal active sites, preventing effective contact with heavy metal ions [29]. As a result, both the utilization efficiency of active functional groups and the overall adsorption performance are reduced. For example, Unnithan et al. [30] reported a maximum adsorption capacity of only 12.4 mg/g for Cr(VI) using modified polyacrylamide—a value substantially lower than that of many high-performance adsorbents-highlighting the marked deficiency of linear polymers in adsorption capacity.

To address the low utilization efficiency of active sites in linear polymer adsorbents, researchers have proposed various strategies to enhance their performance. The two most common approaches are (i) increasing the abundance of active functional groups (e.g., -NH_2_, -COOH) through grafting or copolymerization and (ii) enlarging the specific surface area by constructing porous structures or nanoscale morphologies to expose more adsorption sites [31,32,33,34]. In recent years, hyperbranched polymers have attracted considerable attention in adsorbent design owing to their unique three-dimensional topological structure [35,36]. Hyperbranched polymers possess numerous modifiable terminal active functional groups along their molecular chains, as well as internal cavities [37]. These features not only provide abundant adsorption sites but also promote the diffusion and mass transfer of heavy metal ions, thereby offering significant structural advantages over linear polymers. For instance, Cui et al. [38] used a branched polyethyleneimine-functionalized adsorbent to treat Cu(II) in water. Their results showed that this adsorbent exhibited superior adsorption capacity and a higher adsorption rate for Cu(II) compared with conventional linear polymer adsorbents, confirming the beneficial role of hyperbranched structures in heavy metal removal.

In light of the above research background, this study selected IA-HBP (itaconic anhydride-hyperbranched polymer) as the comonomer. The P(IA-HBP-AA-AM)/MMT composite was successfully prepared via in situ polymerization using acrylic acid (AA), acrylamide (AM), and montmorillonite (MMT). This material combines the high-density active sites of the hyperbranched polymer, the excellent water solubility and complexation ability of the poly(acrylic acid)/polyacrylamide backbone, and the high specific surface area and layered structural stability of MMT, thus demonstrating considerable promise for the efficient capture of heavy metal ions. The composite was subsequently employed to remove heavy metals from mining-contaminated water. Its performance was systematically evaluated with respect to static adsorption toward Pb(II) (including adsorption kinetics, isotherms, and thermodynamic parameters), dynamic adsorption performance (column breakthrough curve and saturation capacity), and selective adsorption ability in the presence of competing metal ions (Cd(II), Zn(II), Al(III), Fe(III), K(I), Na(I)). The objective of this work is to provide an economical, practical, and high-performance adsorbent for the efficient separation and removal of Pb(II) from mining-contaminated water, as well as to offer a theoretical foundation and experimental reference for the application of hyperbranched polymers in the remediation of heavy metal pollution.

## 2. Experimental

### 2.1. Materials

All chemical reagents used in this study are analytical grade reagents. Itaconic acid (IA) was supplied by Alfa Chemical Co., Ltd. (Zhengzhou, China), and was used as received. Ethylene dicarboxylic acid (EDA) was purchased from Kelong Chemical Reagent Corporation (Chengdu, China) and was used without further purification. N,N-Dimethylformamide (DMF), Acrylamide (AM) and Lead nitrate procured from Sinopharm Chemical Reagent Co., Ltd. (Beijing, China), and was used as received. Triethanolamine (TA) procured from Guangdong Guanghua Sci-Tech Co., Ltd. (Guangdong, China), and was used as received. Montmorillonite (MMT) was purchased from Chengdu Aike Chemical Reagent Co., Ltd. (Chengdu, China). Deionized water was used in all preparations.

### 2.2. Equipment

Fourier transform infrared (FTIR) spectra were recorded using a Nicolet 6700 spectrometer (Thermo Fisher Scientific, Waltham, MA, USA). ^1^H-NMR spectra were acquired on a Bruker Ascend 600 spectrometer (Bruker, Rheinstetten, Germany). Scanning electron microscopy (SEM) images were obtained using a SU8010 field emission scanning electron microscope (Hitachi, Ltd., Tokyo, Japan). Transmission electron microscopy (TEM) images were captured using a JEM-2100 transmission electron microscope (JEOL Ltd., Tokyo, Japan). Nitrogen adsorption–desorption isotherms were measured using a ASAP 2020 surface area and porosity analyzer (Micromeritics Instrument Corporation, Norcross, GA, USA) to determine the specific surface area and pore size distribution. Thermogravimetric analysis (TGA) was performed using a STA 449 F3 thermogravimetric analyzer (Netzsch-Gerätebau GmbH, Selb, Germany). The concentration of Pb(II) was determined by inductively coupled plasma optical emission spectrometry (ICP-OES) using an Optima 8000 spectrometer (PerkinElmer, Inc., Waltham, MA, USA). X-ray photoelectron spectroscopy (XPS) measurements were performed using an ESCALAB 250Xi X-ray photoelectron spectrometer (Thermo Fisher Scientific, Waltham, MA, USA). X-ray diffraction (XRD) patterns were recorded using a D8 Advance X-ray diffractometer (Bruker AXS GmbH, Karlsruhe, Germany) with Cu Kα radiation (λ = 1.5406 Å).

### 2.3. Characterization

The chemical structure of the IA-HBP copolymer monomer and the P(IA-HBP-AA-AM)/MMT composite was analyzed by Fourier transform infrared (FTIR) spectroscopy using a Nicolet 6700 spectrometer over the wavenumber range of 400–4000 cm^−1^ with a resolution of 4 cm^−1^. ^1^H nuclear magnetic resonance (^1^H-NMR) spectra were recorded on a Bruker Ascend 600 spectrometer at 600 MHz, using DMSO-d_6_ as the solvent and tetramethylsilane (TMS) as the internal standard. The molecular weight of IA-HBP was determined by gel permeation chromatography (GPC) on an Agilent 1260 Infinity system with tetrahydrofuran (THF) as the mobile phase at a flow rate of 1.0 mL/min, calibrated with polystyrene standards.

The morphology of the composite before and after Pb(II) adsorption was observed using a SU8010 field emission scanning electron microscope at an accelerating voltage of 5 kV. Prior to SEM observation, samples were sputter-coated with gold. Transmission electron microscopy (TEM) images were captured with a JEM-2100 microscope at an acceleration voltage of 200 kV; samples were prepared by dispersing the composite powder in ethanol and dropping onto a copper grid.

Nitrogen adsorption–desorption isotherms were measured at 77 K using an ASAP 2020 surface area and porosity analyzer. The specific surface area was calculated by the Brunauer–Emmett–Teller (BET) method, and the pore size distribution was derived from the desorption branch using the Barrett-Joyner-Halenda (BJH) method. The total pore volume was taken at a relative pressure (P/P_0_) of 0.99. Thermogravimetric analysis (TGA) was performed on a STA 449 F3 analyzer under a nitrogen atmosphere from 25 °C to 800 °C at a heating rate of 10 °C/min. X-ray diffraction (XRD) patterns were recorded on a D8 Advance diffractometer using Cu Kα radiation at 40 kV and 40 mA, with a scan range of 2θ = 10–80° and a step size of 0.02°.

X-ray photoelectron spectroscopy (XPS) measurements were carried out on an ESCALAB 250Xi spectrometer with monochromatic Al Kα X-ray source (hν = 1486.6 eV). The binding energies were calibrated using the C 1s peak at 284.8 eV. The concentration of Pb(II) and other metal ions in solution was determined by inductively coupled plasma optical emission spectrometry (ICP-OES) using an Optima 8000 spectrometer.

### 2.4. Experimental Procedures

#### 2.4.1. Preparation of P(IA-HBP-AA-AM)/MMT Composite

**Figure 1:**
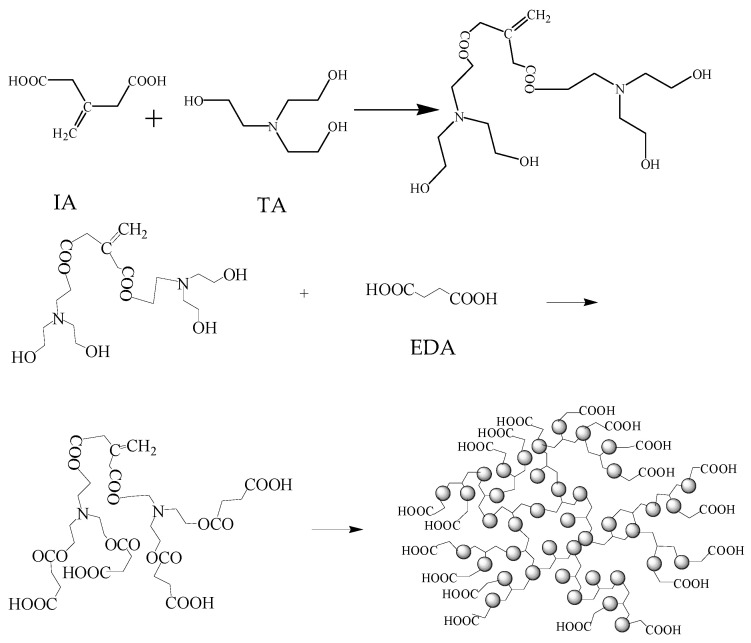
Synthetic route of the IA-HBP copolymer monomer.

Under N_2_ atmosphere, 0.1 mol of itaconic acid and a certain amount of triethanolamine were added to a three-necked flask equipped with a mechanical stirrer and a reflux condenser. Mechanical stirring was initiated and maintained at 250 rpm. Subsequently, 60 mL of DMF was added, and stirring was continued until all reactants were dissolved. The temperature was then raised to 125°C, and the reaction was allowed to proceed for 3.5 h. After that, an appropriate amount of ethylene dicarboxylic acid was introduced into the flask, and the temperature was further increased to 130 °C for an additional 4 h, during which the reaction mixture turned yellow. The resulting product was then concentrated by rotary evaporation at 105 °C, followed by vacuum drying for 24 h to obtain the IA-HBP copolymer monomer. The reaction scheme is illustrated in Figure 1.

Certain amounts of the IA-HBP copolymer monomer, 10.0 g of AA, 4.0 g of AM, and 2.0 g of MMT were dissolved in 30 mL of distilled water and transferred into a three-necked flask. The mixture was subjected to ultrasonic vibration and mechanical stirring for 15 min to obtain a homogeneous dispersion. The mixture was then continuously stirred and heated to 72 °C, after which 1.5 g of N,N′-methylenebisacrylamide was added. After 10 min, a certain amount of potassium persulfate solution was added. The temperature was raised to 75 °C, and the reaction was allowed to proceed until a gel formed. The gel was kept at this temperature for another 1.5 h, and then the system was cooled down. The obtained product was washed and dried to yield the P(IA-HBP-AA-AM)/MMT composite, which was then ground, sieved, and stored for further use.

#### 2.4.2. Static Adsorption Experiments

##### Single-Component Adsorption Experiments

A (0.10 ± 0.005) g aliquot of the P(IA-HBP-AA-AM)/MMT composite was placed in a 250 mL conical flask, and 100 mL of 0.01 mol/L Pb(II) solution was added. The mixture was shaken in a thermostatic shaker under different experimental parameters (adsorption time, pH, and temperature). Subsequently, the mixture was filtered through a 0.22 μm membrane filter (aqueous), and the concentration of Pb(II) in the filtrate was determined by inductively coupled plasma optical emission spectrometry (ICP-OES).

##### Adsorption Isotherm Experiments

Portions of (0.10 ± 0.005) g of the P(IA-HBP-AA-AM)/MMT composite were transferred into 250 mL conical flasks. Subsequently, Pb(II) solutions with varying initial concentrations (C_0_) of 0.001, 0.003, 0.005, 0.008, 0.01, 0.02, 0.03, 0.05, and 0.08 mol/L were added. The mixtures were shaken in a thermostatic shaker under optimal reaction conditions and then filtered through 0.22 μm aqueous membrane filters. The Pb(II) concentration in the filtrate was measured by inductively coupled plasma optical emission spectrometry (ICP-OES).

##### Multicomponent Selective Adsorption Experiments

To mimic the complex composition of real mining-contaminated water, a multi-metal-ion solution was prepared in the laboratory based on typical water quality data from mining areas. To investigate the influence of the P(IA-HBP-AA-AM)/MMT composite on the removal efficiency of Pb(II) from simulated wastewater containing multiple coexisting metal ions, a Pb(II) solution with an initial concentration of 75 mg/L was supplemented with varying concentrations of Cd(II), Zn(II), Al(III), Fe(III), K(I), and Na(I) solutions. Following adsorption equilibrium, the concentrations of the respective heavy metal ions in the filtrate were measured using ICP-OES. Subsequently, the adsorption capacity and adsorption distribution ratio were calculated.

##### Desorption and Reuse Studies

A (0.10 ± 0.005) g aliquot of the P(IA-HBP-AA-AM)/MMT composite was placed in a 250 mL conical flask, and 100 mL of 0.01 mol/L Pb(II) solution was added to initiate adsorption. The Pb(II)-saturated composite was then desorbed using 3% HNO_3_, followed by regeneration with 0.1 mol/L NaOH solution and rinsing with distilled water until neutral. This adsorption–desorption procedure was repeated for five consecutive cycles.

##### Translation of the Dynamic Adsorption Experiment

A total of 1.00 g of P(IA-HBP-AA-AM)/MMT composite was homogeneously packed into a polyvinyl chloride (PVC) column. The Pb(II) solution was introduced from the bottom to the top using a peristaltic pump at a flow rate of 0.30 mL/min. Effluent fractions were collected, and the Pb(II) concentration in each fraction was measured. The ratio of the effluent concentration (C_t_) to the initial concentration (C_0_) was plotted as a function of the effluent volume (V), enabling the calculation of the dynamic adsorption capacity.

## 3. Result and Discussion

### 3.1. Structural Analysis of IA-HBP Copolymer Monomer

**Figure 2:**
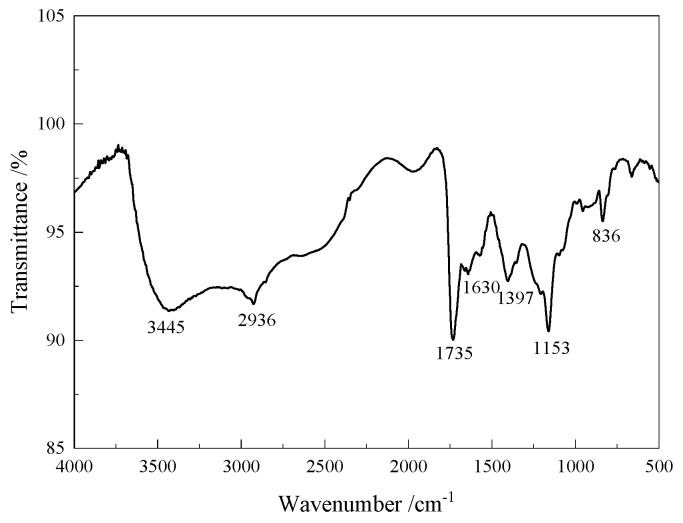
FTIR spectrum of IA-HBP.

Figure 2 presents the FTIR analysis results of the IA-HBP copolymer monomer, revealing key features of its chemical structure. As shown, the strong absorption peaks at 3445 cm^−1^ and 1735 cm^−1^ correspond to the stretching vibrations of O-H and C=O in the carboxylic acid groups, respectively, indicating that the surface of the IA-HBP copolymer monomer is rich in carboxyl functional groups. The absorption peak at 1630 cm^−1^ is attributed to the characteristic stretching vibration of C=C, confirming the presence of carbon-carbon double bonds in the product structure. The absorption peak at 1153 cm^−1^ corresponds to the C-N stretching vibration, indicating that triethanolamine has successfully reacted with itaconic acid (IA). In addition, the absorption peak at 2936 cm^−1^ is assigned to the asymmetric stretching vibration of C-H in methylene groups, while the characteristic peak at 1397 cm^−1^ arises from the bending vibration of -CH_2_- in succinic acid. Combined with relevant literature [39], these data collectively demonstrate that the IA-HBP copolymer monomer has been successfully prepared.

**Figure 3:**
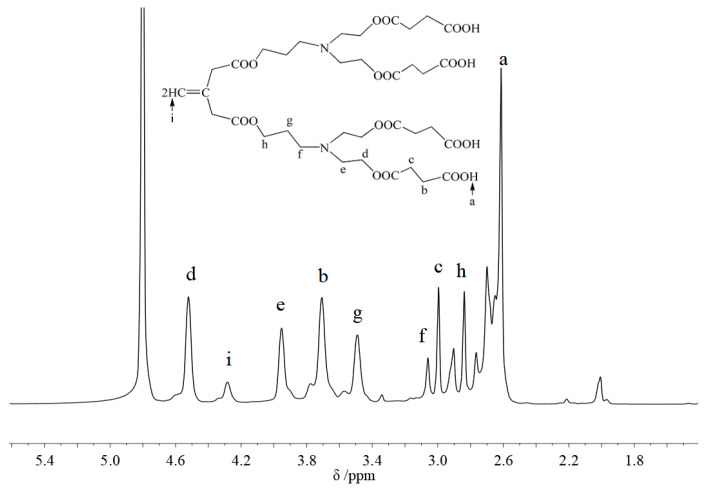
^1^H-NMR spectrum of IA-HBP.

**Figure 4:**
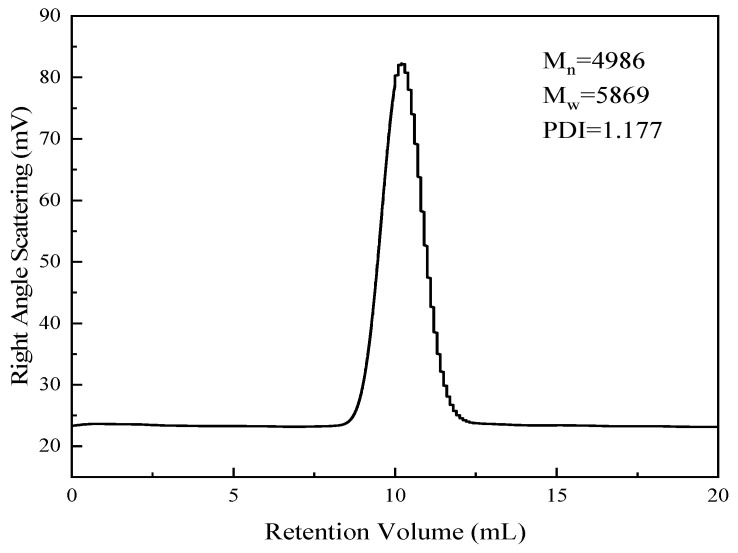
The GPC analysis of IA-HBP.

The ^1^H-NMR spectrum of the IA-HBP copolymer monomer is shown in Figure 3. The spectral assignments are as follows: the signal at δ = 2.65 ppm is attributed to the protons of the terminal carboxylic acid groups; the peak at δ = 2.8 ppm indicates the esterification reaction between itaconic acid and triethanolamine; and the peak at δ = 3.0 ppm corresponds to the methylene protons adjacent to the carbonyl group in succinic acid. And more, the molecular weight of IA-HBP was characterized by GPC, with the results presented in Figure 4. The number-average molecular weight (M_n_) and weight-average molecular weight (M_w_) were determined to be 4986 and 5869, respectively, yielding a polydispersity index (PDI) of 1.177. Taken together, the FT-IR, ^1^H-NMR, and GPC results demonstrate the successful synthesis of IA-HBP copolymer monomer.

### 3.2. Structural Analysis of P(IA-HBP-AA-AM)/MMT Composite

**Figure 5:**
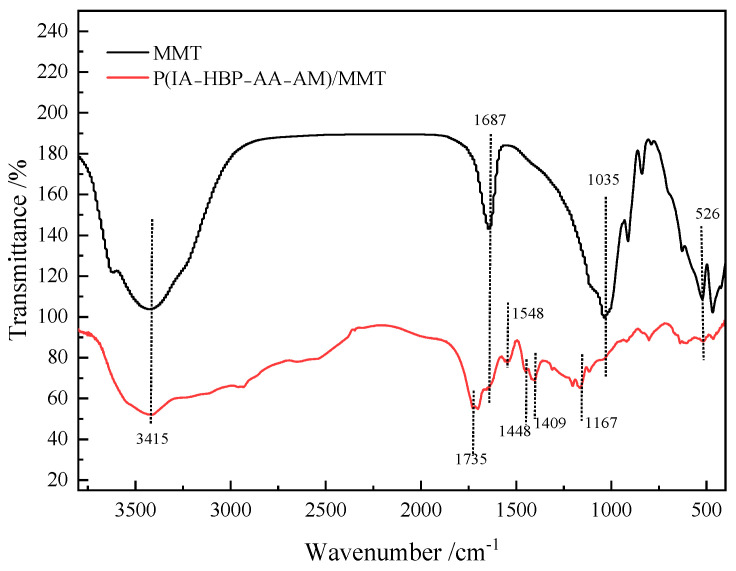
FTIR spectrum of P(IA-HBP-AA-AM)/MMT composite.

Figure 5 shows the FTIR spectrum of the MMT and P(IA-HBP-AA-AM)/MMT composite adsorbent material. As can be seen, a broad and blunt strong absorption peak appears at 3415 cm^−1^, which corresponds to the stretching vibration of -OH. The broad shape of this peak indicates the presence of a large number of associated hydroxyl groups, mainly derived from the carboxyl groups in the polymer network and possibly adsorbed water molecules. Two distinct characteristic absorption peaks appear at 1735 cm^−1^ and 1687 cm^−1^, both attributed to the stretching vibration of carbonyl (C=O). The presence of these two peaks proves the existence of a large number of ester (-COO-) or free carboxyl (-COOH) functional groups in the material structure. These oxygen-containing groups can serve as coordination sites for metal ions, providing abundant active centers for the subsequent adsorption of Pb(II). The absorption peak at 2928 cm^−1^ corresponds to the asymmetric stretching vibration of methylene (CH_2_-), indicating that the polymer backbone contains aliphatic carbon chain structures. Moreover, a distinct absorption peak is observed in the range of 1600–1650 cm^−1^, indicating that all unsaturated C=C bonds in the comonomers (IA-HBP, AM, AA) have fully participated in the polymerization reaction, confirming the successful copolymerization of the three components. In addition, in the low-wavenumber region, the absorption peaks at 1035 cm^−1^ and 526 cm^−1^ can be assigned to the characteristic stretching and bending vibrations of Si-O-Si or Si-O-R [40]. Meanwhile, the sharp absorption peak of MMT originally present at 3624 cm^−1^ (caused by interlayer free water) has completely disappeared. This change indicates that the interlayer environment of MMT has been altered, and chemical bonding has formed between the hydroxyl groups on its surface or edges and the polymer chains, proving that montmorillonite has been successfully grafted onto the IA-HBP-AA-AM polymer network, forming an organic-inorganic hybrid structure.

### 3.3. Thermal Performance Analysis of P(IA-HBP-AA-AM)/MMT Composite

**Figure 6:**
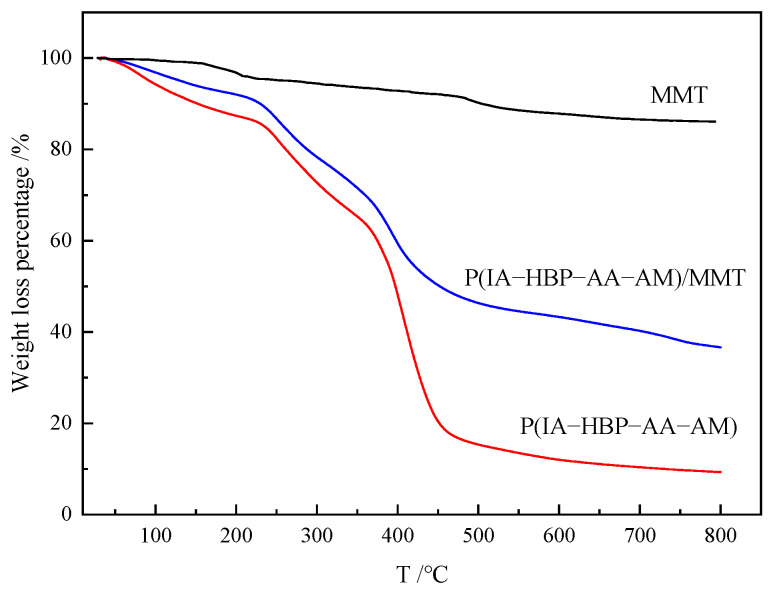
TGA curves of IA-HBP-AA-AM and P(IA-HBP-AA-AM)/MMT composite.

The thermogravimetric analysis (TGA) curves of the MMT, IA-HBP-AA-AM copolymer and the P(IA-HBP-AA-AM)/MMT composite are shown in Figure 6. Both materials display a three-stage weight loss profile. The first stage, spanning from 120 to 190 °C, is primarily attributable to the evaporation of adsorbed free water and a minor amount of bound water. The second stage, from 190 to 450 °C, exhibits the most pronounced weight loss, which is mainly due to the thermal decomposition of oxygen-containing functional groups (e.g., carboxyl, ester, and hydroxyl groups) and partial carbonization of the polymer chains. The third stage occurs above 450 °C, where residual carbonaceous species are further oxidized and evolve as CO_2_, leading to a progressive mass reduction. A comparative analysis of the two TGA curves indicates that the final residual mass of the P(IA-HBP-AA-AM)/MMT composite is markedly higher than that of the IA-HBP-AA-AM copolymer without MMT. This enhancement arises from the inherent thermal stability of montmorillonite, whose layered structure serves as a physical barrier and thermal insulator at high temperatures, thereby delaying the thermal degradation of the polymer matrix and significantly improving the overall thermal stability of the composite.

### 3.4. XRD Analysis of P(IA-HBP-AA-AM)/MMT Composite

**Figure 7:**
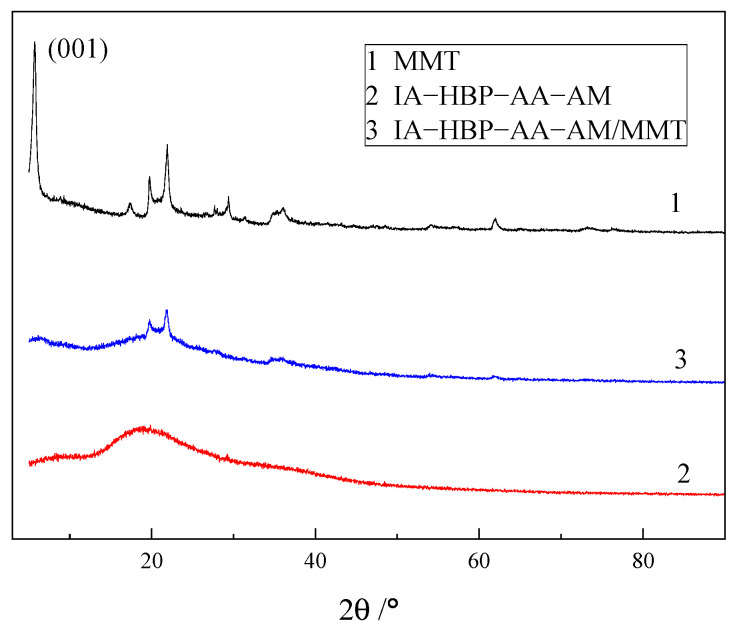
X-ray diffraction pattern of P(IA-HBP-AA-AM)/MMT composite.

**Figure 8:**
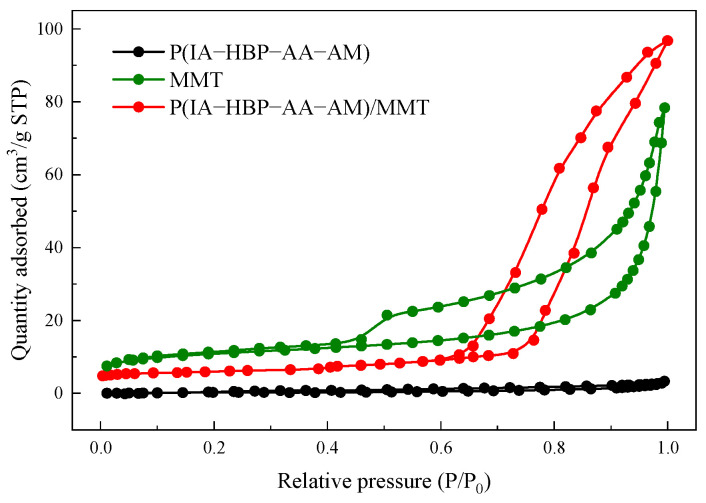
N_2_ adsorption–desorption isotherm of P(IA-HBP-AA-AM)/MMT composite.

**Figure 9:**
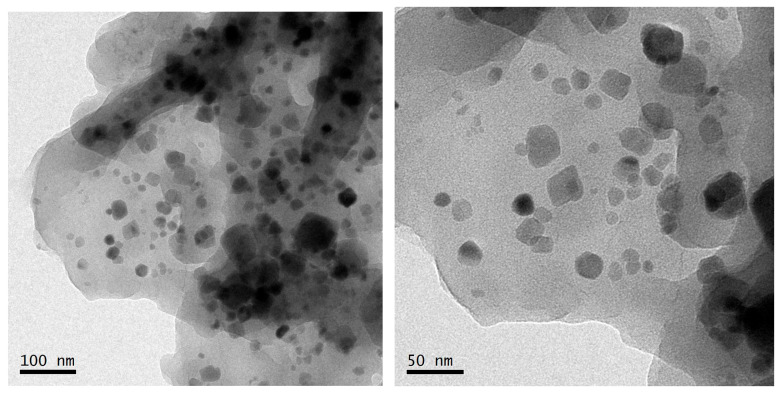
TEM images of P(IA-HBP-AA-AM)/MMT composite.

The XRD patterns of the P(IA-HBP-AA-AM)/MMT composite are shown in Figure 7. As seen in curve 1, pristine MMT displays a narrow and sharp characteristic diffraction peak at 2θ = 5.8°, which is assigned to the (001) crystal plane and reflects its ordered layered structure [41]. In contrast, after introducing MMT into the IA-HBP-AA-AM polymer network, the characteristic diffraction peak at 2θ = 5.8° in its XRD pattern almost completely disappears, with only a broad, halo-like diffuse peak appearing at around 2θ = 20.5°. The former indicates that the original ordered layered structure of MMT is destroyed: during the rapid exothermic polymerization, the heat generated and the intercalation of polymer chains jointly promote the exfoliation of MMT. After exfoliation, the nanolayers no longer maintain long-range ordered stacking but are uniformly dispersed in a disordered state within the polymer matrix, leading to the disappearance of the characteristic peak. The latter (the diffuse peak at 2θ ≈ 20.5°) is typical of amorphous or highly disordered structures, mainly attributed to the highly branched topology of the IA-HBP-AA-AM polymer and the asymmetric arrangement of its molecular chains, which hinder the formation of regular crystalline regions and thus produce typical amorphous diffuse scattering. This also indirectly confirms the formation of the hyperbranched structure and its uniform distribution in the composite. To further support the XRD results, specific surface area data (Figure 8) are considered: pristine MMT has a specific surface area of 39.3 m^2^/g, the polymer only 0.9 m^2^/g, while the composite exhibits 26.6 m^2^/g, still close to that of pristine MMT. This indicates that the layered surface of MMT remains largely exposed after compositing, which is possible only when MMT is exfoliated into nanolayers and uniformly dispersed; if only intercalation or agglomeration had occurred, the specific surface area would be significantly reduced. Meanwhile, TEM images (Figure 9) directly confirm that the MMT nanolayers are distributed in a disordered, isolated state within the polymer matrix, with no stacked agglomerates observed. In summary, the disappearance of the characteristic diffraction peak in the XRD pattern can be attributed to the complete exfoliation of MMT promoted by the exothermic polymerization and polymer chain intercalation. The exfoliated nanolayers are randomly dispersed in the hyperbranched polymer network, failing to form long-range ordered diffraction, thus resulting in an exfoliated nanocomposite.

The nitrogen adsorption–desorption isotherm of the MMT, IA-HBP-AA-AM copolymer and the P(IA-HBP-AA-AM)/MMT composite are shown in Figure 8. As presented in Figure 8, the P(IA-HBP-AA-AM)/MMT composite is typical of a Type IV isotherm. The hysteresis loop in the mid-relative-pressure region is indicative of capillary condensation occurring in the porous adsorbent. Additionally, the specific surface area of the composite is 26.6 m^2^/g, which lies between that of pure MMT (39.3 m^2^/g) and the pure polymer P(IA-HBP-AA-AM) (0.9 m^2^/g), and is closer to the value of MMT. This indicates that the polymer is successfully loaded onto the surface or into the interlayer spaces of MMT, covering or blocking some of the adsorption sites, resulting in a decrease of about 32% in specific surface area compared to pure MMT. However, because the polymer does not completely encapsulate all MMT lamellae and micropores, a considerable proportion of the MMT surface remains accessible to nitrogen molecules; therefore, the specific surface area of the composite is still much higher than the extremely low value of the pure polymer (0.9 m^2^/g). Additionally, the material exhibits a mesoporous structure with a total pore volume of 12.1197 cm^3^/g and a narrow pore size distribution centered at approximately 32.53 nm, which is favorable for Pb(II) diffusion and adsorption. These results suggest that the composite forms a nanostructure with partial polymer intercalation or surface coating, achieving organic-inorganic phase combination while retaining a certain adsorption capacity.

TEM images (Figure 9) show that the MMT nanolayers present as thin, dark ribbon-like or lamellar structures with sizes of several tens of nanometers. These nanolayers are uniformly dispersed in a disordered, isolated state within the P(IA-HBP-AA-AM) polymer matrix, with no obvious stacked agglomerates or multilayer parallel arrangements observed. This morphological feature is highly consistent with the complete disappearance of the MMT (001) characteristic diffraction peak (2θ = 5.8°) in the XRD pattern.

### 3.5. Analysis of Adsorption Behavior in a Single-Component System

#### 3.5.1. Effect of Contact Time on Adsorption Capacity

**Figure 10:**
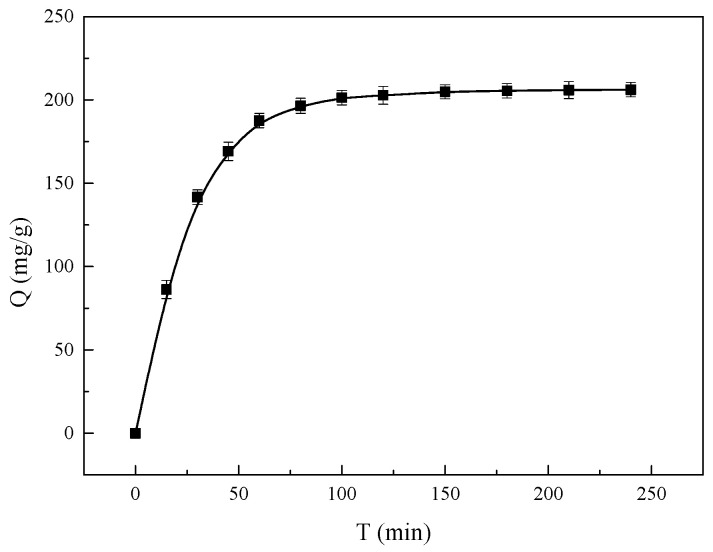
Effect of adsorption time on Pb(II) adsorption onto P(IA-HBP-AA-AM)/MMT composite.

Figure 10 shows the effect of contact time on the efficiency of Pb(II) removal by P(IA-HBP-AA-AM)/MMT composite. As can be seen, the adsorption rate of Pb(II) onto P(IA-HBP-AA-AM)/MMT composite is the fastest within 0–60 min, and the adsorption capacity increases rapidly. This is because at the initial stage of adsorption, a large number of unoccupied active sites (such as carboxyl, ester and other oxygen-containing functional groups) exist on the surface of the composite material. Pb(II) can quickly bind to these sites, and the adsorption process is mainly controlled by external surface diffusion and surface complexation, representing a typical fast external adsorption stage. During the adsorption stage from 60 to 150 min, as the adsorption time increases, the easily accessible active sites on the material surface are gradually occupied by Pb(II), and the number of available vacancies significantly decreases. Therefore, although the adsorption capacity continues to increase, the adsorption rate declines markedly. At this stage, the dominant mechanism shifts to intra-particle diffusion: Pb(II) must migrate from the material surface into the internal pores or the polymer network, where the mass transfer resistance increases, leading to a slower adsorption rate [42]. Finally, at 150 min, the adsorption capacity no longer changes significantly with time, reaching adsorption equilibrium, with an equilibrium adsorption capacity of 204.9 mg/g. This result indicates that there is strong electrostatic attraction and coordination between P(IA-HBP-AA-AM)/MMT composite and Pb(II), enabling the material to achieve efficient removal of Pb(II) within a relatively short time.

**Figure 11:**
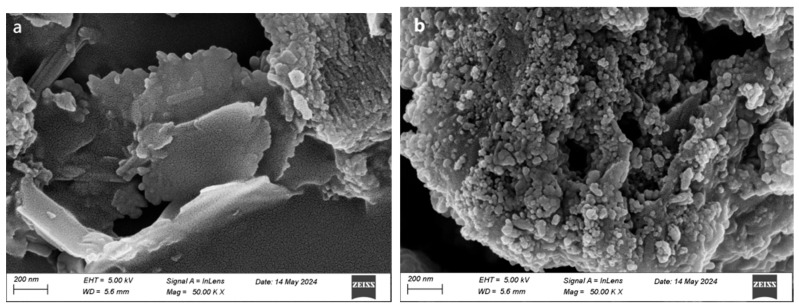
SEM images of P(IA-HBP-AA-AM)/MMT before (**a**) and after (**b**) Pb(II) adsorption.

Figure 11a,b show the SEM images of P(IA-HBP-AA-AM)/MMT composite before and after Pb(II) adsorption. It can be seen that before adsorption (Figure 11a), the material surface is relatively smooth and flat, exhibiting a relatively uniform morphology after compounding the polymer matrix with montmorillonite, with no obvious granular deposits. After adsorption (Figure 11b), however, the surface becomes significantly rougher, with numerous irregular protrusions, folds, or granular agglomerates. The main reason for this morphological change is that during the adsorption process, strong coordination or electrostatic interactions occur between Pb(II) ions and the abundant oxygen-containing functional groups (such as carboxyl -COOH and hydroxyl -OH) on the material surface. As adsorption proceeds, Pb(II) is gradually captured and deposited on the composite surface, forming a deposit layer or aggregated structure composed of lead species, thereby transforming the surface microstructure from smooth and flat to rough and uneven. The significant difference in surface morphology visually confirms the effective adsorption and immobilization of Pb(II) by P(IA-HBP-AA-AM)/MMT composite, and also reflects the full physicochemical interaction between the active sites on the material surface and the heavy metal ions.

**Figure 12:**
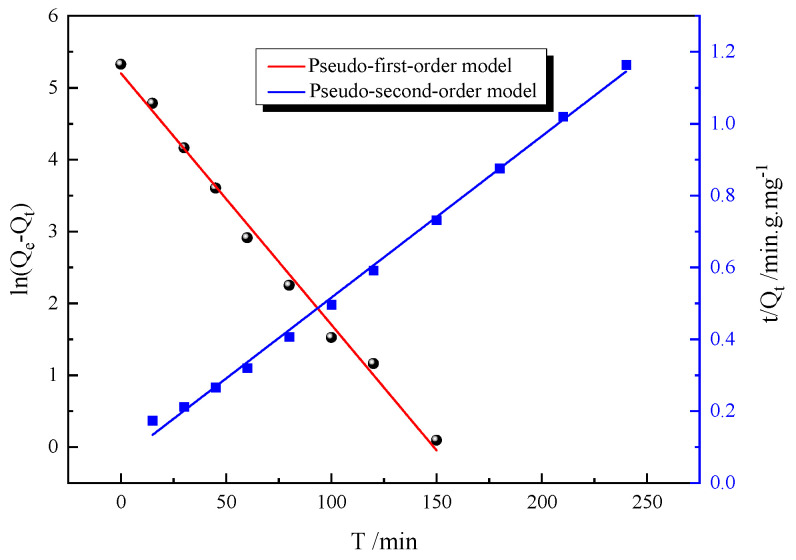
Linear fitting curves of pseudo-first-order and pseudo-second-order kinetics for Pb(II) adsorption onto P(IA-HBP-AA-AM)/MMT composite.

To further clarify the adsorption process, the obtained adsorption data were fitted using two kinetic models: the pseudo-first-order and pseudo-second-order models [43,44]. The results are shown in Figure 12, and the calculated kinetic parameters are presented in Table 1. The results indicate that the pseudo-second-order kinetic model provides a higher correlation coefficient (R^2^ = 0.997) for fitting the Pb(II) adsorption onto P(IA-HBP-AA-AM)/MMT composite, and its calculated equilibrium adsorption capacity is closer to the experimental value. This suggests that the adsorption behavior of Pb(II) by P(IA-HBP-AA-AM)/MMT composite is better described by the pseudo-second-order model, implying that the adsorption occurs via chemisorption involving covalent bonding or electron exchange interactions between P(IA-HBP-AA-AM)/MMT composite and Pb(II).

#### 3.5.2. Effect of pH on Adsorption Capacity

**Figure 13:**
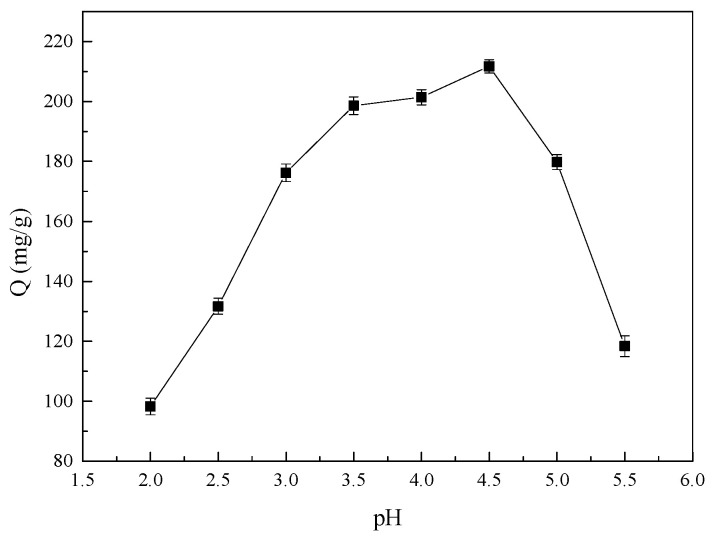
Effect of pH on Pb(II) adsorption onto P(IA-HBP-AA-AM)/MMT composite.

According to the literature [45], the pH of the adsorption environment directly affects the protonation degree of the surface functional groups of P(IA-HBP-AA-AM)/MMT composite, and ultimately influences its effectiveness in adsorbing Pb(II). Figure 13 shows the effect of pH on Pb(II) adsorption onto P(IA-HBP-AA-AM)/MMT composite. It can be seen that the equilibrium adsorption capacity of the P(IA-HBP-AA-AM)/MMT composite for Pb(II) at different pH values is generally greater than 100 mg/g, indicating that the material has a broad pH applicability for Pb(II) adsorption. When the adsorption environment is strongly acidic, the presence of a large amount of H_3_O^+^ in the solution causes the -COOH and -CONH_2_ groups in P(IA-HBP-AA-AM)/MMT to become protonated, thereby weakening the electrostatic attraction and chelation ability between P(IA-HBP-AA-AM)/MMT composite and Pb(II) to varying degrees [46]. As the pH of the adsorption environment increases, the competitive effect between H_3_O^+^ and Pb(II) diminishes. Meanwhile, the protonated -COOH and -CONH_2_ groups on the surface of P(IA-HBP-AA-AM)/MMT composite begin to deprotonate, enhancing the electrostatic attraction between the active sites on the material surface and Pb(II) [47]. Consequently, the adsorption capacity of P(IA-HBP-AA-AM)/MMT composite for Pb(II) gradually increases, reaching a maximum adsorption value of 211.7 mg/g at pH = 4.5. In addition, when pH ≥ 5, OH^−^ and Pb(II) tend to form Pb(OH)_2_ precipitates in the aqueous solution, leading to a decrease in the adsorption capacity of P(IA-HBP-AA-AM)/MMT composite. Therefore, pH = 4.5 was selected for subsequent studies.

#### 3.5.3. Effect of Temperature on Adsorption Behavior

**Figure 14:**
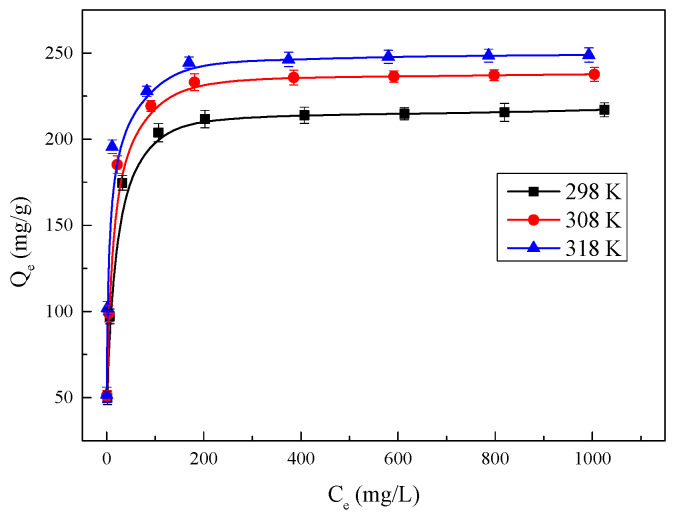
Effect of temperature on Pb(II) adsorption onto P(IA-HBP-AA-AM)/MMT composite.

To clarify the adsorption behavior between P(IA-HBP-AA-AM)/MMT composite and Pb(II), the effect of temperature on the Pb(II) adsorption performance of P(IA-HBP-AA-AM)/MMT composite was further investigated. The results are shown in Figure 14. The adsorption capacity of P(IA-HBP-AA-AM)/MMT composite for Pb(II) first increases rapidly with increasing initial concentration and then tends to level off. This is mainly because the higher concentration gradient of Pb(II) provides a greater driving force for adsorption. Moreover, as the adsorption temperature increases, the equilibrium adsorption capacity of P(IA-HBP-AA-AM)/MMT composite for Pb(II) increases slightly, indicating that a higher temperature favors the adsorption process.

**Figure 15:**
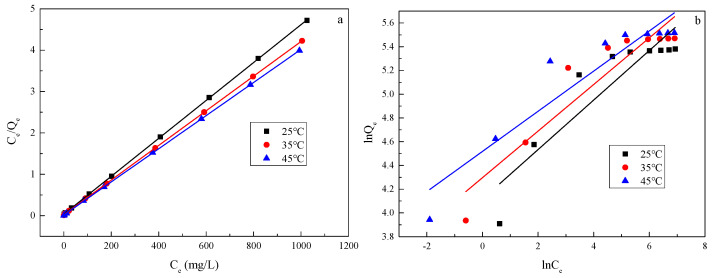
Linear fitting curves of the Langmuir (**a**) and Freundlich (**b**) adsorption isotherm models for Pb(II) adsorption onto P(IA-HBP-AA-AM)/MMT composite.

Furthermore, the Langmuir and Freundlich adsorption isotherm models were used to evaluate the adsorption behavior of Pb(II) onto P(IA-HBP-AA-AM)/MMT composite [48,49,50]. The fitting curves are shown in Figure 15, and the calculated parameters are listed in Table 2. The Langmuir isotherm model provides a higher correlation coefficient for fitting the Pb(II) adsorption onto P(IA-HBP-AA-AM)/MMT composite, indicating that the adsorption behavior follows the Langmuir isotherm model more closely. This suggests that the adsorption of Pb(II) onto P(IA-HBP-AA-AM)/MMT composite likely occurs as monolayer adsorption on a homogeneous surface. In addition, Furthermore, as the temperature increases, the maximum adsorption capacity of P(IA-HBP-AA-AM)/MMT composite for Pb(II) increases from 218.34 mg/g (298 K) to 249.38 mg/g (298 K), indicating that a higher temperature is favorable for the adsorption of Pb(II) by P(IA-HBP-AA-AM)/MMT.

Meanwhile, the comparison with other adsorbent materials in Table 3 demonstrates that the P(IA-HBP-AA-AM)/MMT composite exhibits significant advantages in Pb(II) adsorption capacity and kinetics, proving its high feasibility for Pb(II) adsorption in aqueous solutions.

Based on the above experimental results, the thermodynamic behavior and parameters of Pb(II) adsorption onto P(IA-HBP-AA-AM)/MMT composite at different temperatures were further investigated. The results are shown in Table 4. In general, ΔG^0^ < 0 indicates that the Pb(II) adsorption process is spontaneous, and its absolute value increases slightly with increasing temperature, suggesting that the spontaneous tendency of the adsorption process becomes stronger. Moreover, the values of ΔH^0^ (9.99 kJ/mol) and ΔS^0^ (39.35 J/(mol·K)) are both > 0, indicating that the adsorption of Pb(II) by P(IA-HBP-AA-AM)/MMT composite is an endothermic reaction accompanied by an increase in randomness. In summary, these results demonstrate that the adsorption process of Pb(II) by this material is a spontaneous endothermic reaction.

#### 3.5.4. Static Adsorption and Regeneration of Pb(II) by P(IA-HBP-AA-AM)/MMT Composite

**Figure 16:**
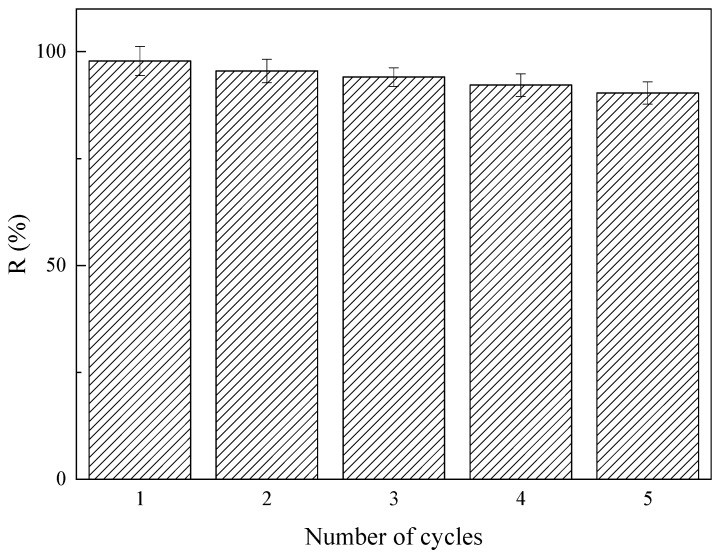
Adsorption–desorption cycles of Pb(II) onto P(IA-HBP-AA-AM)/MMT composite (C_0_ = 75 mg/L, pH = 4.5, T = 298 K).

As shown in Figure 16, after five cycles of performance testing, the removal rate of Pb(II) by P(IA-HBP-AA-AM)/MMT composite remains close to 90%. The loss in removal efficiency is mainly attributed to incomplete desorption, where some Pb(II) still occupies the adsorption sites; additionally, about 5% fine mass loss during the experimental recovery process leads to a reduction in adsorption sites. Given the above results, the removal rate of Pb(II) by P(IA-HBP-AA-AM)/MMT composite remains relatively stable, indicating that this adsorbent has good reusability in Pb(II) adsorption experiments.

### 3.6. Adsorption Mechanism

**Figure 17:**
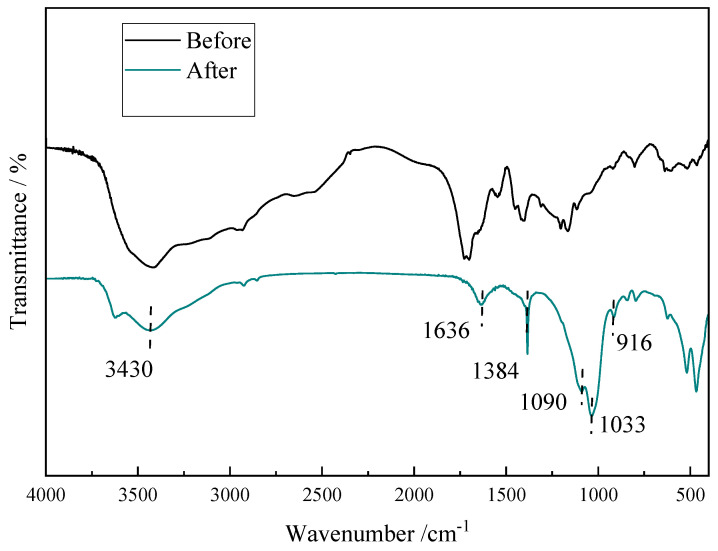
FTIR spectra of P(IA-HBP-AA-AM)/MMT composite before and after Pb(II) adsorption.

As shown in Figure 17, a comparative analysis of the infrared spectra of the P(IA-HBP-AA-AM)/MMT composite before and after Pb(II) adsorption reveals significant changes in both the positions and intensities of the characteristic absorption peaks, confirming that effective chemical adsorption of Pb(II) occurs on the adsorbent surface. Before adsorption, the composite displays a broad, blunt O-H stretching vibration peak at 3415 cm^−1^, attributed to carboxyl, hydroxyl, and adsorbed water. The peaks at 1735 cm^−1^ and 1687 cm^−1^ correspond to free carboxylic C=O and hydrogen-bonded C=O, respectively. The bands at 1035 cm^−1^ and 526 cm^−1^ are assigned to the stretching and bending vibrations of the montmorillonite framework (Si-O-Si). After Pb(II) adsorption, these peaks undergo systematic changes: the O-H stretching peak blue-shifts to 3430 cm^−1^, indicating that hydroxyl groups participate in coordinating with Pb(II), thereby altering the electron cloud density of the O-H bond and the hydrogen-bonding network. The two C=O absorption peaks originally at 1735 cm^−1^ and 1687 cm^−1^ disappear completely, while a new broad peak emerges at 1636 cm^−1^, assigned to the superposition of the asymmetric stretching vibration of carboxylate and the amide I band. More importantly, a distinct new peak appears at 1384 cm^−1^, attributed to the symmetric stretching vibration of carboxylate coordinated with Pb(II) to form a -COO-Pb inner-sphere complex. This change directly demonstrates that a large number of carboxyl groups on the adsorbent surface undergo deprotonation during adsorption and bind to Pb(II) via ionic or coordination bonds, serving as the primary driving force for chemical adsorption, which is consistent with the pseudo-second-order kinetic model. In addition, the characteristic framework peak of montmorillonite shifts only slightly from 1035 cm^−1^ to 1033 cm^−1^, and new peaks at 1090 cm^−1^ and 916 cm^−1^ (assigned to Al-OH bending vibrations) appear, indicating a slight adjustment of the interlayer environment due to cation exchange. Nevertheless, the Si-O framework remains intact, suggesting that the structure of montmorillonite as a support is not destroyed.

**Figure 18:**
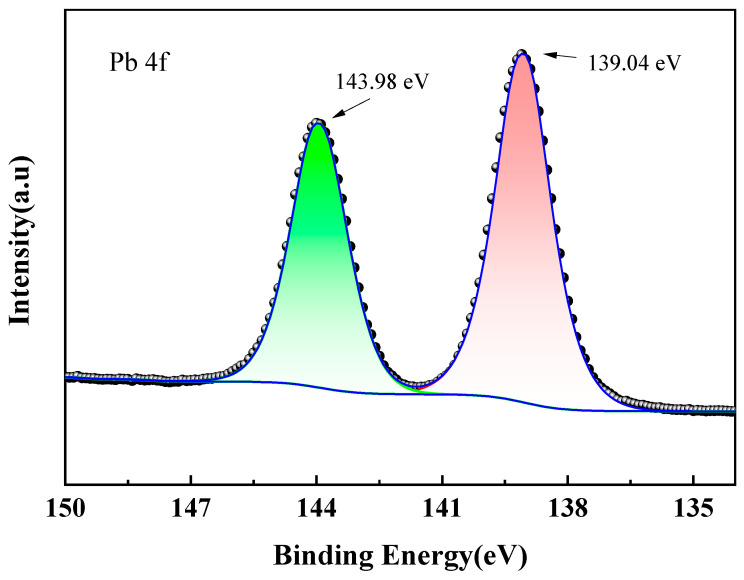
XPS of Pb 4f spectrum of P(IA-HBP-AA-AM)/MMT after Pb(II) adsorption.

Figure 18 presents the XPS spectrum of the P(IA-HBP-AA-AM)/MMT composite after lead adsorption. The corresponding Pb 4f photoelectron peak is observed at a binding energy of 139.04 eV, a feature characteristic of lead carbonate and lead oxalate compounds, suggesting that lead is bound to the composite via hydroxyl and carbonyl groups [56]. Moreover, the single symmetrical peak at 139.04 eV indicates that the lead species immobilized on the composite surface exists solely in the divalent oxidation state.

### 3.7. Adsorption Selectivity

The selectivity experiments were conducted using a laboratory-prepared multi-metal-ion solution whose composition (Pb(II), Cd(II), Zn(II), Al(III), Fe(III), K(I), and Na(I)) was designed to reflect typical mining-impacted waters, as detailed in Multicomponent Selective Adsorption Experiments Section. Therefore, it is crucial to further investigate the effect of coexisting ions on Pb(II) adsorption in mining area contaminated water. Table 5 shows the selective adsorption performance of P(IA-HBP-AA-AM)/MMT composite for Pb(II) under multi-ion coexistence conditions. As can be seen from Table 5, the P(IA-HBP-AA-AM)/MMT composite exhibits excellent selective adsorption performance for Pb(II) under multi-ion coexistence conditions. Except for Zn(II) and Cd(II), whose adsorption efficiencies are 6.75% and 9.75%, respectively, the adsorption efficiencies for other coexisting ions (Al(III), Fe(III), K(I), Na(I)) are all below 5%. This is mainly attributed to differences in physicochemical properties such as ionic radius and hydration energy, and also indicates that the material possesses high selectivity for Pb(II). To further elucidate the origin of this high selectivity, we systematically analyzed the material’s structural characteristics. First, FTIR (Figure 5) confirms that the composite surface is rich in -COOH and -OH groups. According to the hard-soft acid-base (HSAB) principle, Pb(II) as a soft Lewis acid exhibits strong affinity toward soft bases such as -COO^−^ and -O^−^, whereas the coexisting ions (Al(III), Fe(III), Zn(II), Cd(II)) are harder or borderline acids, leading to weaker interactions. XPS analysis (Figure 18) further reveals a clear shift in the Pb 4f binding energy after adsorption, confirming the coordination between Pb(II) and -COO^−^. Second, electrostatic attraction at the working pH plays a key role: as shown in Figure 13, the Pb(II) adsorption capacity increases sharply in the pH range of 2.0–4.5, indicating that deprotonation of -COOH and -OH enhances the negative surface charge. At pH = 4.5 (the condition used for selectivity tests), the composite surface is negatively charged, strongly attracting Pb(II) cations while showing weak interaction or repulsion toward other cations. Third, the hyperbranched topology and MMT exfoliation also contribute to the selectivity: the three-dimensional hyperbranched structure (IA-HBP) provides internal cavities and numerous terminal groups, increasing the local concentration of binding sites. Meanwhile, the exfoliated MMT layers (confirmed by XRD in Figure 7 and TEM in Figure 9) create a high-aspect-ratio, negatively charged surface that acts as a “molecular sieve” for Pb(II), excluding larger hydrated ions or those with lower affinity (e.g., Al(III) and Fe(III) may be partially hindered). Finally, ion exchange also plays a role: the presence of exchangeable cations (K(I), Na(I)) in the system (Table 4) suggests that ion exchange between Pb(II) and these monovalent cations contributes to selectivity. In summary, the multiple synergistic mechanisms described above collectively endow the P(IA-HBP-AA-AM)/MMT composite with excellent selectivity for Pb(II).

**Figure 19:**
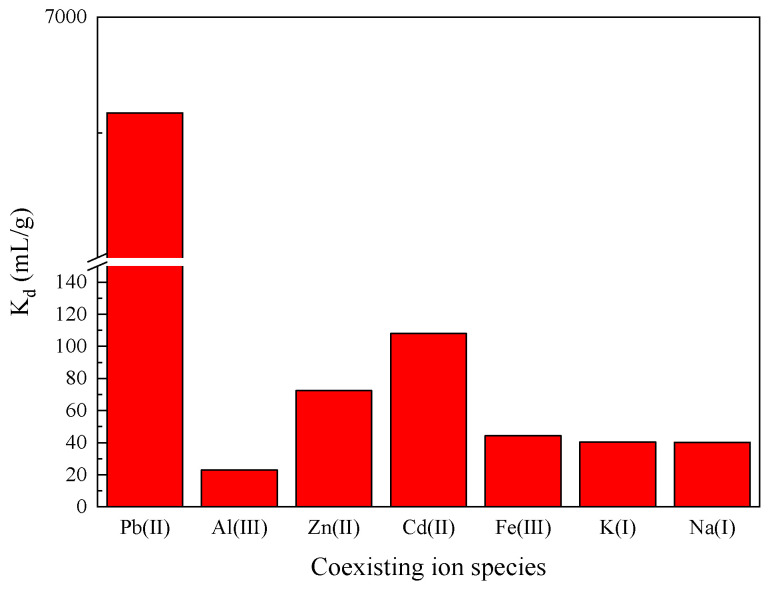
Adsorption distribution ratios (K_d_) of various ions by P(IA-HBP-AA-AM)/MMT composite in simulated mining area contaminated water.

The adsorption distribution ratios (K_d_) of all coexisting ions in the simulated mining area contaminated water are shown in Figure 19. As can be seen from Figure 16, under multi-cation coexistence conditions, the K_d_ of Pb(II) remains at a high level (6583 mL/g) and is significantly higher than that of other ions, further demonstrating that P(IA-HBP-AA-AM)/MMT possesses high adsorption selectivity for Pb(II).

### 3.8. Dynamic Adsorption Analysis

**Figure 20:**
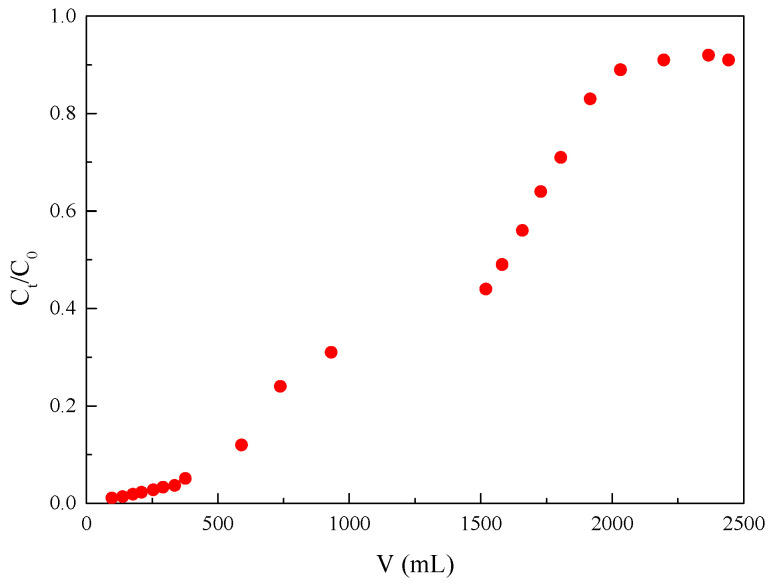
Breakthrough curve of Pb(II) onto P(IA-HBP-AA-AM)/MMT composite.

To further illustrate the adsorption characteristics of P(IA-HBP-AA-AM)/MMT composite for Pb(II), dynamic adsorption experiments were carried out under the conditions of C_0_ = 500.0 mg/L, m = 1.0 g, and v = 1.60 mL/min. The results are shown in Figure 20. As can be seen from the figure, when the breakthrough rate of Pb(II) in the P(IA-HBP-AA-AM)/MMT composite column reached 5%, the treated solution volume was approximately 376.4 mL. When the influent solution volume reached 1519 mL, the column reached adsorption saturation. The corresponding adsorption capacity at c_t_/c_0_ = 0.05 and the total adsorption capacity were 178.7 mg/g and 435.8 mg/g, respectively. The utilization rate of the ion exchange column, calculated as the ratio of these two values, was approximately 41%, indicating that the as-prepared P(IA-HBP-AA-AM)/MMT composite adsorbent exhibits good adsorption performance for Pb(II) in simulated mining area contaminated water [57].

## 4. Conclusions

In this study, a novel hyperbranched polymer-based composite, P(IA-HBP-AA-AM)/MMT, was successfully synthesized via in situ polymerization. The incorporation of montmorillonite (MMT) not only enhanced the thermal stability of the composite, but also contributed to a specific surface area of 26.6 m^2^/g, providing a favorable platform for adsorption. Static adsorption experiments demonstrated that the composite exhibits excellent Pb(II) uptake, with the adsorption kinetics following the pseudo-second-order model and the isotherm data fitting the Langmuir model, indicating chemisorption and monolayer coverage. Thermodynamic analysis confirmed that the adsorption is endothermic and spontaneous. Under optimized conditions (pH = 4.5, 45 °C), the maximum adsorption capacity reached 249.38 mg/g, outperforming many conventional adsorbents. More importantly, the composite showed remarkable selectivity for Pb(II) in the presence of competing ions (Cd(II), Zn(II), Al(III), Fe(III), K(I), Na(I)), with a high distribution coefficient (K_d_ = 6583 mL/g), and maintained approximately 90% of its removal efficiency after five adsorption–desorption cycles, demonstrating good reusability. Dynamic column experiments further revealed a saturation adsorption capacity of 178.7 mg/g and a column utilization efficiency of 41%, highlighting its practical potential for treating Pb(II)-contaminated wastewater. Collectively, these findings establish P(IA-HBP-AA-AM)/MMT as a cost-effective, highly selective, and regenerable adsorbent for the efficient removal of Pb(II) from complex mining-impacted waters and industrial effluents.

## Figures and Tables

**Table 1:** Kinetic parameters for Pb(II) adsorption onto P(IA-HBP-AA-AM)/MMT composite.

C_0_ (mg/L)	Pseudo-First-Order Rate Parameter			Pseudo-Second-Order Rate Parameter		
	Q_e_ (mg/g)	k (1/min)	R^2^	K_t_ (g/(mg·min))	Q_e_ (mg/g)	R^2^
2072	181.88	0.035	0.992	0.000306	222.22	0.997

**Table 2:** Parameters of the adsorption isotherm models for Pb(II) adsorption onto P(IA-HBP-AA-AM)/MMT composite.

T (°C)	Langmuir Constants			Freundlich Constants		
	Q_m_ (mg/g)	K_L_ (L/mg)	R^2^	K_F_ (L/g)	n^−1^	R^2^
25	218.34	0.133	0.998	61.36	0.209	0.8101
35	238.67	0.183	0.999	73.48	0.196	0.8617
45	249.38	0.275	0.997	91.65	0.169	0.8709

**Table 3:** Comparison of adsorption performances for Pb(II) on other materials.

Materials	Capacity, Q_max_ (mg/g)	Kinetics (min)	Ref.
MoS_2_/CeO_2_	99.87	40	[51]
Corncobs	8.28	20	[52]
W-MoS_2_@CA	160.2	120	[53]
MoS_2_/Fe_3_O_4_	199.3	180	[54]
MMT K_10_	40.86	68	[55]
P(IA-HBP-AA-AM)/MMT	249.38	60	This work

**Table 4:** Thermodynamic parameters for Pb(II) adsorption onto P(IA-HBP-AA-AM)/MMT composite at different temperatures.

T (°C)	lnK^0^ (kJ/mol)	ΔG^0^ (kJ/mol)	ΔH^0^ (kJ/mol)	ΔS^0^ (J/(mol∙k))
25	0.647	−1.603	9.99	39.35
35	0.871	−2.231		
45	1.012	−2.676		

**Table 5:** Adsorption rates of P(IA-HBP-AA-AM)/MMT composite for various ions in simulated mining area contaminated water.

Ion Species	Initial Concentration/(mg/L)	Supernatant Concentration/(mg/L)	Adsorption Efficiency/%
Pb(II)	75	9.89	86.81
Al(III)	120	117.3	2.33
Zn(II)	80	74.6	6.75
Cd(II)	40	36.1	9.75
Fe(III)	160	153.2	4.25
K(I)	10	9.61	3.9
Na(I)	150	144.2	3.87

## Data Availability

The data used to support the findings of this study are available from the corresponding author upon request due to privacy reasons.
